# Thermodynamics and Equations of State of Iron to 350 GPa and 6000 K

**DOI:** 10.1038/srep41863

**Published:** 2017-03-06

**Authors:** P. I. Dorogokupets, A. M. Dymshits, K. D. Litasov, T. S. Sokolova

**Affiliations:** 1 Institute of Earth’s Crust, SB RAS, Irkutsk, Russia; 2 V. S. Sobolev Institute of Geology and Mineralogy SB RAS, Novosibirsk, Russia; 3 Novosibirsk State University, Novosibirsk, Russia

## Abstract

The equations of state for solid (with bcc, fcc, and hcp structures) and liquid phases of Fe were defined via simultaneous optimization of the heat capacity, bulk moduli, thermal expansion, and volume at room and higher temperatures. The calculated triple points at the phase diagram have the following parameters: bcc–fcc–hcp is located at 7.3 GPa and 820 K, bcc–fcc–liquid at 5.2 GPa and 1998 K, and fcc–hcp–liquid at 106.5 GPa and 3787 K. At conditions near the fcc–hcp–liquid triple point, the Clapeyron slope of the fcc–liquid curve is d*T*/d*P* = 12.8 K/GPa while the slope of the hcp–liquid curve is higher (d*T*/d*P* = 13.7 K/GPa). Therefore, the hcp–liquid curve overlaps the metastable fcc–liquid curve at pressures of about 160 GPa. At high-pressure conditions, the metastable bcc–hcp curve is located inside the fcc-Fe or liquid stability field. The density, adiabatic bulk modulus and P-wave velocity of liquid Fe calculated up to 328.9 GPa at adiabatic temperature conditions started from 5882 K (outer/inner core boundary) were compared to the PREM seismological model. We determined the density deficit of hcp-Fe at the inner core boundary (*T* = 5882 K and *P* = 328.9 GPa) to be 4.4%.

Iron is a major component of the Earth’s core, therefore knowledge of its *P–V–T* relations and thermodynamic properties is extremely important^1^,^2^,^3^,^4^,^5^,^6^. The phase diagram of Fe is relatively complex due to the existence of several polymorphic modifications^7^,^8^. At the standard conditions (*T* = 298.15 K and *P* = 1 bar), iron is a ferromagnet and has a body-centred cubic (bcc) structure (α-Fe or bcc-Fe). The Curie temperature (*T*_C_) of 1043 K marks the transition to the paramagnetic state with the same structure. At this transition, the heat capacity of Fe has a characteristic λ-shape form with a maximum at *T*_C_^9^,^10^. At 1185–1667 K the crystal structure of iron changes to a face-centred cubic (fcc) cell (γ-Fe or fcc-Fe), however, above 1667 K and up to the melting temperature of 1811 K, iron again has the bcc structure (δ-Fe).

At 10.5 GPa and 753 K^7^ (or by more precise and recent measurements at 8.2 GPa and 678 K^11^), there is a triple point between bcc-Fe, fcc-Fe, and the high-pressure phase hcp-Fe, which has the hexagonal close-packed (hcp) structure (hcp-Fe). It was argued that hcp-Fe is likely a stable phase in the inner core of the Earth^12^,^13^,^14^. However, bcc-Fe is also suggested as a reliable candidate in the inner core^15^,^16^. There are two triple points along the melting line of Fe. The bcc-Fe and fcc-Fe phases are stable with liquid at the first triple point at *Р* = 5.2 GPa and *T* = 1991 K^7^, whereas hcp-Fe and fcc-Fe were found in equilibrium with liquid at the second triple point, whose location is variable in different works (e.g. *Р* = 88 GPa and *Т* = 2800 K^17^ or *Р* = 98.5 GPa and *Т* = 3712 K^14^).

The most recent thermodynamic calculations of the Fe phase diagram including solid phases and liquid were performed in a few studies^18^,^19^,^20^,^21^ using the CALPHAD thermodynamic formalism^10^,^22^. In these calculations, the volume contribution to the Gibbs energy was calculated using the Anderson–Grüneisen parameter^23^. In addition, a wide-range multiphase equation of state (EoS) to 10 TPa and 10^5^ K was calculated^24^.

During the last several years, a significant amount of new *P–V–T* data for fcc-Fe and hcp-Fe, especially at very high temperatures, have appeared^6^,^13^,^25^,^26^,^27^,^28^ and, in addition, the melting curve of Fe was shifted to higher temperatures according to measurements in ref. 14. In these works, various pressure scales were used based on the EoSs of Au, MgO, NaCl, KCl, and hcp-Fe. Thus, the problem of consistent EoSs for solid phases (bcc-Fe, fcc-Fe, and hcp-Fe) and liquid iron remains extremely important. These EoSs should be consistent with the phase diagram of iron, including the melting curve and with *P–V–T*, thermochemical, and sound velocity data at 0.1 MPa.

In this work, we propose updated EoSs for Fe phases based on a formalism from our recent publications^29^,^30^,^31^,^32^,^33^,^34^,^35^,^36^. In addition, the magnetic contribution to the Helmholtz free energy was calculated according to ref. 10 and 37, whereas the EoS of Fe liquid was calculated using the standard approach^38^,^39^,^40^.

## Thermodynamic model for solid and liquid iron

The Helmholtz free energy of solid phases can be expressed in its classical form^41^ as:


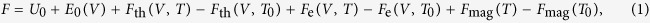


where *U*_0_ is the reference energy, *E*_0_(*V*) is the potential (cold) part of the free energy at the reference isotherm *T*_0_ = 298.15 K, which depends only on *V, F*_th_(*V, T*) is the thermal part of the free energy, which depends on *V* and *T, F*_e_(*V, T*) is the free electrons’ contribution to the Helmholtz free energy, which also depends on *V* and *Т*, and *F*_mag_(*T*) is the magnetic contribution, depending on *T* only.

The pressure at 298 K isotherm is calculated from the Vinet–Rydberg equation^42^:


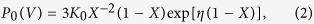


where *X* = (*V*/*V*_0_)^1/3^, and *η* = 3*K*_0_′/2 − 3/2. Differentiating eq. (2) with respect to volume, we obtain the bulk modulus at reference isotherm *T*_0_ = 298.15 K and its pressure derivative:


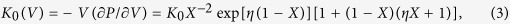



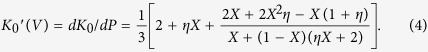


The potential energy at the 298 K isotherm is:


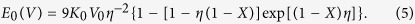


The thermal part of the Helmholtz free energy can be expressed via the Einstein model, which coincides with the Debye model at the high-temperature limit^43^:


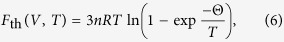


where Θ is the characteristic temperature depending on volume, which is related to the Debye temperature (Θ_D_) via Θ = 0.75 Θ_D_; *n* is the number of atoms in the chemical formula of the compound, and *R* is the gas constant.

Differentiating eq. (6) with respect to temperature at constant volume one can obtain the entropy, the thermal part of the free energy and the heat capacity at constant volume:


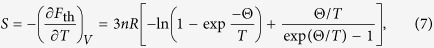



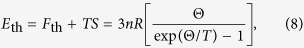



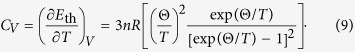


Differentiating eq. (6) with respect to volume at constant temperature, one can obtain the thermal pressure and isothermal bulk modulus:


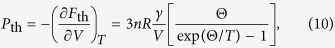



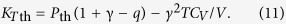


In eqs (10, 11) *γ* is the Grüneisen parameter, 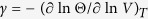, and 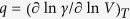. Differentiation of the thermal pressure with respect to temperature at constant volume gives the pressure slope at constant volume:


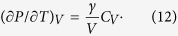


The volume dependence of *γ* and *q* was accepted in the Altshuler form^44^:


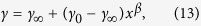



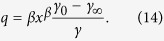


The volume dependence of the Einstein temperature can be expressed as:


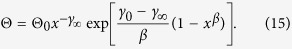


In eqs (12,13,14,15) *γ*_0_ is the Grüneisen parameter at reference conditions, *γ*_∞_ is the Grüneisen parameter at infinite compression (*x* = 0), and β is a fitted parameter.

The contribution of free electrons to the Helmholtz free energy in the simplest form can be expressed as^41^:


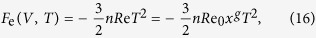


where the parameter *e*_0_ denotes the electronic contribution to the Helmholtz free energy, *g* is an electronic analogue of the Grüneisen parameter, and *x* = *V*/*V*_0_. The contribution of this part to entropy, internal energy, heat capacity, pressure, isothermal bulk modulus, and pressure slope at constant volume can be estimated as:


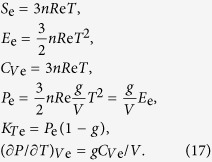


It should be emphasized that if the electronic contribution to the Helmholtz free energy is not equal to zero, the thermal Grüneisen parameter:


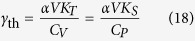


will be different from the Grüneisen parameter calculated from eq. (13).

The formalism for the magnetic contribution to the Helmholtz free energy was adapted from ref. 10 and 37 with modifications from ref. 45 to obtain the correct limit of entropy at 0 K. The magnetic contribution to the Helmholtz free energy can be expressed as^45^:


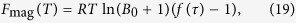


where *B*_0_ is an average magnetic moment per atom, τ = *T*/T*, T* is the critical temperature (*T*_C_ for ferromagnetic materials and Neel temperature *T*_N_ for paramagnetic materials). The magnetic moment^10^,^37^ of bcc-Fe is *B*_0_ = 2.22. The function *f*(τ) can be written as:


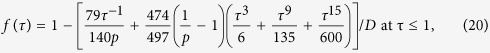



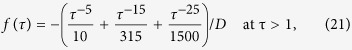



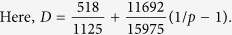


The value of the parameter *p* changes from *p* = 0.4 (for bcc-Fe) to *p* = 0.28 for other Fe polymorphs. However, it was shown that at 18 GPa bcc-Fe transforms into hcp-Fe and the value of the magnetic moment becomes zero^46^. Based on another study^47^, the magnet moment of both fcc-Fe and hcp-Fe approaches zero with increasing temperature and pressure. In our study, the EoS of fcc-Fe and hcp-Fe were constrained with the magnetic moment fixed to zero and this assumption is common for thermodynamic calculations of the iron phase diagram at high pressures^6^,^18^,^19^,^20^,^21^,^48^,^49^.

The equation for the Helmholtz free energy for liquid metal can be rewritten^38^,^39^,^40^. The entropic parameter *a*_*S*_, which characterizes residual entropy of the liquid at 0 K is introduced. Accordingly, in equation (1) for liquid Fe, the magnetic part is excluded, whereas the entropy parameter is added:


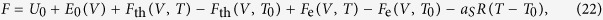


The reference temperature for liquid Fe at 0.1 MPa is *T*_0_ = 1811 K. Along the reference isotherm, the pressure was calculated using eq. (2). In the first approximation, the entropic parameter is independent of volume. Calibration of fitted parameters for liquid iron is considered below.

The full solution of the equations allowed us to find all necessary parameters for the thermodynamic description of the EoS for a solid phase. In addition, from the obtained parameters one can calculate the coefficient of thermal expansion *α* = (∂*P*/∂*T*)_*V*_/*K*_*T*_, heat capacity at constant pressure *C*_*P*_ = *C*_*V*_ + *α*^2^*TVK*_*T*_, and adiabatic bulk modulus *K*_*S*_ = *K*_*T*_ + *VT*(*αK*_*T*_)^2^/*C*_*V*_, which can also be compared with direct experimental measurements. The enthalpy and the Gibbs free energy can be found from the following relations: *H* = *E* + *PV, G* = *F* + *PV*.

## EoSs for solid and liquid Fe to 350 GPa

The fitted parameters of the EoSs for bcc-Fe, fcc-Fe, hcp-Fe, and liquid iron (Table 1) were obtained by simultaneous optimization of the experimental measurements of the heat capacity, volume and thermal expansion, adiabatic bulk modulus at room pressure, and *P–V–T* data (all references are included in the Supplementary Information). Pressures in the *P–V–T* dataset were corrected based on the self-consistent ruby pressure scale or EoSs of Au, MgO, and W^31^,^35^,^36^. Supplementary Figures S1–S4 show the temperature dependence of the isobaric heat capacity, the volume coefficient of thermal expansion, molar volume, adiabatic and isothermal bulk modulus for bcc-Fe, fcc-Fe, and hcp-Fe at 0.1 MPa calculated from our EoSs in comparison with direct experimental measurements and reference data. Supplementary Figures S5–S8 show the differences between calculated pressures at different temperatures with direct experimental measurements of pressure. The pressure scales are shown in the legends to the figures.

### Body-centred cubic iron (bcc-Fe)

Supplementary Figure S5 shows that our EoS for bcc-Fe is reliably consistent with experimental measurements^49^,^50^,^51^, which were obtained in quasihydrostatic conditions in He or Ne pressure media. The pressure in these works was calculated using ruby^30^,^35^ and Au^52^ pressure scales and in the pressure range of 0–15 GPa these scales give consistent results. The measurements in Ne and Ar pressure media show an alternative deviation from our data^53^. In another paper^54^, the measurement was performed in non-hydrostatic conditions and deviates from our data at higher pressures. In ref. 55 and 56, pressure was calculated using the NaCl EoS^57^,^58^. As was shown recently^59^, this scale underestimates pressure by 0.5 GPa (at 10–15 GPa) in comparison with another NaCl EoS^60^. If one recalculates the data from ref. 55, 56 and 60 using the NaCl scale^60^, a better consistency with our EoS for bcc-Fe is obtained, which confirms the relevance of the experimental data mentioned above^55^,^56^. The pressure obtained based on the compressional wave sound velocity and density measurements of bcc-Fe^61^ is also shown in Supplementary Figure S5 for comparison. The calculated thermodynamic properties (*P, T, x* = *V*/*V*_0_, α, *S, C*_*V*_, *C*_*P*_, *K*_*T*_, *K*_*S*_, *γ*_th_, *K*′, and Gibbs energy, *G*) for α-Fe (bcc-Fe) are listed in Supplementary Table S1 (see also bcc-Fe.xls in the Supplementary Information).

### Face-centred cubic iron (fcc-Fe)

The calculated thermodynamic properties for γ-Fe (fcc-Fe) are listed in Supplementary Table S2 (see also fcc-Fe.xls in Supplementary Information). Supplementary Figure S6 shows that our EoS is reliably consistent with experimental *P–V–T* data^26^,^28^,^62^,^63^ calibrated using Au, MgO, and NaCl pressure scales. At high pressures, the present EoS of fcc-Fe averages out the experimental measurements^14^,^17^, where pressures were calculated using the EoS of hcp-Fe^49^. One more experimental dataset^64^ overestimates pressures by up to 6 GPa compared with our data for fcc-Fe. The calculated molar volume of fcc-Fe at 0.1 MPa is consistent with measurements in ref. 65 and 66 (Supplementary Figure S6). The calculated volumes of fcc-Fe^26^,^28^ are plotted between bcc and fcc phases in Supplementary Figure S2. Their isothermal bulk modulus is consistent with our calculations (Supplementary Figure S4). The Grüneisen parameter for fcc-Fe (eq. 13) is almost independent of volume (Table 1); however, the thermal Grüneisen parameter (eq. 18) decreases with increase of temperature and pressure up to 1.5–1.6 at 100 GPa and 3000–4000 K (Supplementary Table S2).

### Hexagonal close-packed iron (hcp-Fe)

The calculated thermodynamic properties for ε-Fe (hcp-Fe) are listed in Table S3 (see also hcp-Fe.xls in Supplementary data). The 298 K isotherm for ε-Fe was calculated using the compressibility curve from ref. 49 corrected based on pressure scales^31^,^35^. The other parameters were calculated by optimization of the *P–V–T* data (Supplementary Figures S7, S8). Most measurements are scattered for less than ±2 GPa in the 80 GPa pressure range (Supplementary Figure S7). However, the more scattered data^62^ deviate by −2 to +4 GPa at 298 K isotherm and significantly overestimate pressures at high temperatures. It can be emphasized that in the Supplementary Figure S8 the measurements for hcp-Fe^14^ deviate in the same way, but with larger scattering at 140 GPa than data for fcc-Fe in Supplementary Figure S6. Two sets of data^13^,^67^ at 250–300 GPa are significantly different (Supplementary Figures S8). This may indicate that either the pressure scales used were incorrect or significant stress was accumulated in the samples.

### Calibration of Gibbs energy of hcp-Fe

**Figure 1:**
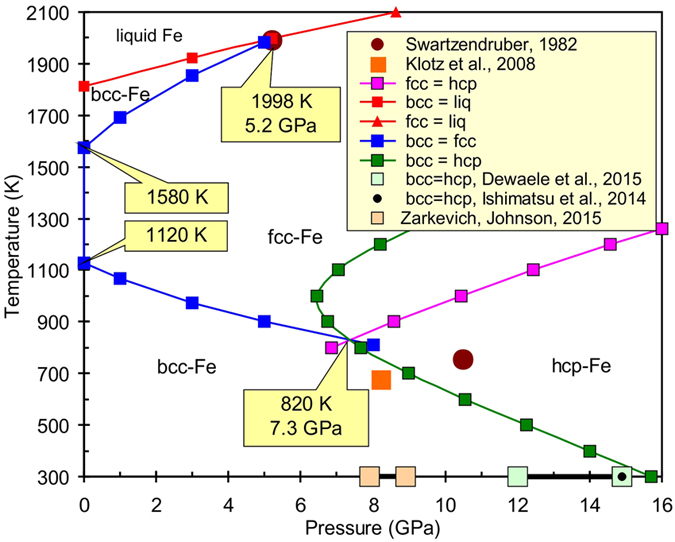
Calculated phase diagram of iron at pressures up to 16 GPa in comparison with reference data^7^,^11^,^68^,^69^,^70^.

The calibration of the Gibbs energy of hcp-Fe was performed using the α–γ–ε triple point^11^, the α–ε transition at room temperature^68^,^69^,^70^, and considering the slope of the γ–ε transition^14^ (Fig. 1). One more important marker for calibration of the Gibbs energy for hcp-Fe and liquid Fe was the γ–ε–liquid triple point^14^. Recently, the *P–V–T* relationships for hcp-Fe in different pressure-transmitting media at 300 K up to 205 GPa and at 1800 K up to 100 GPa have been investigated^6^. To calculate the pressure, the Ne, NaCl-B2, and Pt pressure scales^52^, and the MgO pressure scale^71^ were used. Supplementary Figures S7, S8 show that values obtained in ref. 6 are in reasonable agreement with our EoS of hcp-Fe. The authors^6^ used a third-order Birch–Murnaghan EoS to fit the parameters on the room temperature isotherm with a fixed initial density *ρ*_0_ = 8.2695 g cm^−3^ and obtained *K*_0_ = 172.7 GPa and *K*_0_′ = 4.79. The pressure calculated based on these parameters is 3 GPa higher than that obtained by our EoS at 50–150 GPa. At higher pressures, the room temperature isotherm^6^ is consistent with our EoS of hcp-Fe (Supplementary Figure S8). At the condition of the inner core boundary (~330 GPa and ~6000 K), our EoS is in good agreement with the EoS obtained in ref. 6. These authors determined the density deficit at the inner core boundary^72^ to be 3.6%, whereas based on our data it would be 4.4% at *T* = 5882 K and *P* = 328.9 GPa.

### γ–ε–liquid triple point

**Figure 2:**
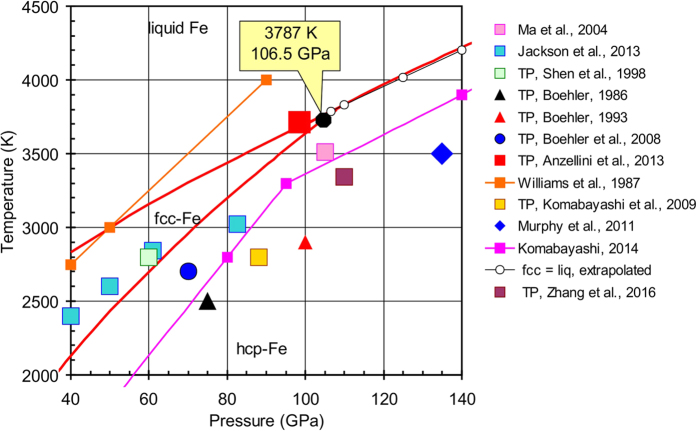
Calculated phase diagram of iron at pressures from 40 to 140 GPa in comparison with reference data^14^,^17^,^20^,^73^,^74^,^75^,^77^,^79^,^80^,^81^,^97^. TP: triple point fcc–hcp–liquid in Fe.

The position of the γ–ε–liquid triple point in the phase diagram of iron has been determined many times and revealed highly controversial results (Fig. 2). The position of the triple point was estimated at 75 GPa and 2500 K^73^. This point was also placed at 100 GPa and 2700 K^1^. Later, the point was shifted back to 75 GPa and 2700 K^74^. Another scientific group^75^ placed the γ–ε–liquid triple point at 60 GPa and 2800 K using pressures calculated from the EoS of hcp-Fe^12^ and EoS of MgO^76^. The melting line of Fe determined in ref. 77 is located at a higher temperature than most determined triple points (Fig. 2). The pressure in this work was estimated before laser heating using the old ruby scale^78^. Melting of Fe was also determined at 105 GPa and 3510 K^79^ with pressures estimated by the EoS of hcp-Fe^12^ before laser heating. Estimations in ref. 17 revealed the triple point at 88 GPa and 2800 K. In addition, the melting temperature of Fe at the core–mantle boundary (*P* = 135 GPa) at 3500 ± 100 K with careful estimation of thermal pressure and anharmonicity was determined^80^. A novel method was presented for detecting the solid–liquid phase boundary of compressed iron at high temperatures using synchrotron Mössbauer spectroscopy^81^. The melting points are shown in Fig. 2 and the pressure was determined using the ruby scale^82^ with corrections for thermal pressure (note that at Fig. 2 their data are shown without these corrections). Finally, the melting line of Fe measured using a synchrotron-based fast X-ray diffraction method was also proposed^14^. The authors fixed the triple point at *P* = 98.5 GPa and *T* = 3712 K. The pressures were estimated from the EoS of hcp-Fe^49^ and KCl^83^. Thus, direct experimental measurements of the melting line of Fe and the γ–ε–liquid triple point indicate about 40 GPa uncertainty in pressure and about 1000 K uncertainty in temperature. The triple point calculated from our data is located at *P* = 106.5 GPa and *T* = 3787 K.

### EoS of liquid iron

**Figure 3:**
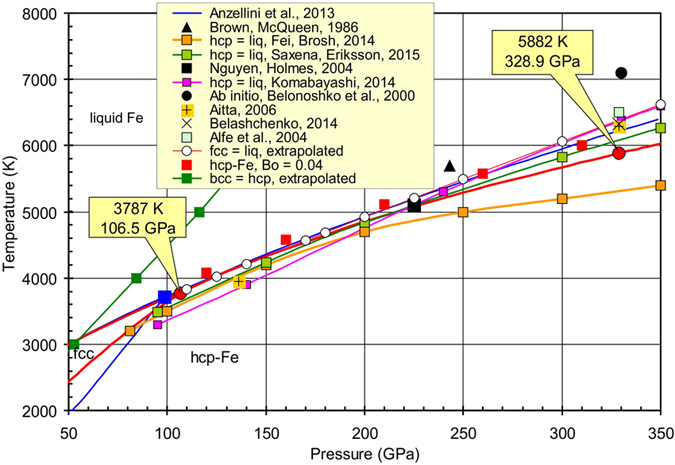
Calculated phase diagram of iron at pressures up to 350 GPa (red solid lines) in comparison with reference data^14^,^19^,^20^,^21^,^90^,^91^,^93^,^94^,^98^,^99^. Curves fcc–liq and bcc–hcp are extrapolated.

The EoS of liquid Fe was estimated using the following approach (eq. 22). Standard conditions for liquid Fe were chosen at *T*_0_ = 1811 K and *P* = 0.1 MPa. For these conditions, the following parameters were recommended^84^: *V*_0_ = 7.957 cm^3^ mol^−1^, *K*_*S*0_ = 109.5 GPa, *α*(*V*) = 92E–6 K^−1^, *γ*_0_ = 1.735, and *C*_*P*_ = 46.632 J mol^−1 ^K^−1^. Fitting parameters for our EoS for liquid iron were calculated using the following assumptions (as a starting point for calculations): (i) the Gibbs energy of α-Fe and liq-Fe must be the same at 1811 K and 0.1 MPa; (ii) the entropy of liquid Fe increases by 7.626 J mol^−1 ^K^−1^ upon melting^9^ in comparison with the entropy of α-Fe; (iii) the melting line of Fe was taken from ref. 14 as more accurate. The fitted parameters of EoS for liquid Fe are listed in Table 1. The calculated thermodynamic properties for liquid Fe are listed in Supplementary Table S4 (see also liquid-Fe.xls in Supplementary Information). The calculated melting lines of iron from the bcc, fcc, and hcp phases are shown in Figs 1, 2 and 3.

The thermodynamic properties of liquid iron at a pressure of 0.1 MPa calculated from our EoS (Supplementary Table S4) are in good agreement with the measured values of density^84^, the sound velocity (*v*_*P*_) and adiabatic bulk modulus^84^,^85^,^86^. The calculated entropy at pressure 0.1 MPa is very close to the reference data^9^.

## Discussion and geophysical implications

We applied the new EoS data for all Fe phases to calculate the phase diagram up to 350 GPa (Figs 1, 2 and 3). Our melting line of Fe is in close agreement with that from ref. 14 because our EoS of liquid iron is based mainly on these data. The calculated triple points have the following parameters: α–γ–liquid is located at 5.2 GPa and 1998 K, α–γ–ε at 7.3 GPa and 820 K, and γ–ε–liquid at 106.5 GPa and 3787 K.

### Melting of iron at high pressure

Most of the previous estimations plot the melting temperature of iron below the melting line obtained from our EoSs (Fig. 3). For example estimations in refs 1 and 18 indicate melting temperatures of 5000 K and 5600 ± 200 K at 330 GPa, respectively. The melting line of Fe in ref. 19 was calculated using the revised thermodynamic properties of Fe. Their triple point γ–ε–liquid is located at 81 GPa and 3200 K and at 330 GPa the melting line is located at 5400 K. Another estimation indicates a melting temperature of 6100 K at 330 GPa^21^. The newly measured melting line of Fe extrapolated to 330 GPa reveals a temperature of about 6230 ± 500 K^14^. Most of the *ab initio* estimations provide higher melting temperatures of Fe at 330 GPa compared with our data: 6700 ± 600 K^87^, 6370 ± 100 K^88^, 6900 ± 400 K^89^, 7100–7200 K^90^, 6325 K^91^, and 6345 K^92^.

**Figure 4:**
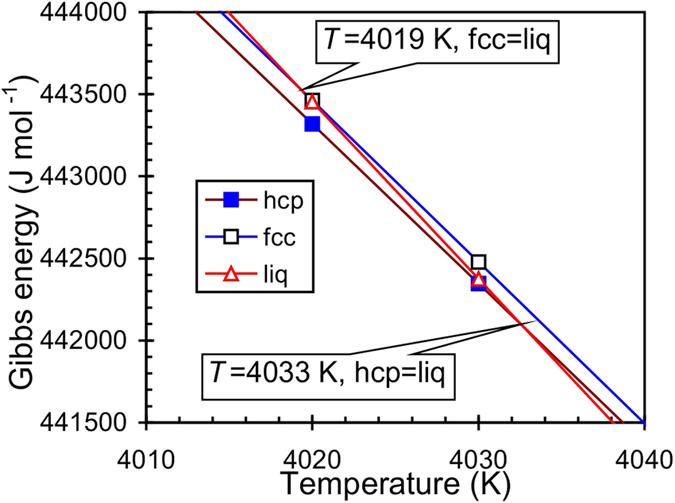
Calculated Gibbs energy of hcp-, fcc-, and liquid Fe at different temperatures at pressure *P* = 125 GPa.

Our thermodynamic calculations indicate a melting temperature of hcp-Fe at 5882 K at 328.9 GPa, and it is in a good agreement with the estimated melting temperature *T* = 5800 ± 500 K obtained from the shock wave data^93^ and extrapolation from the experiments^14^, which is *T* = 6230 ± 500 K. Figures 2 and 3 show the fcc–liquid melting curve and its extrapolation to high pressures. The melting curve was found to be very close to the hcp–liquid curve. At the conditions near the fcc–hcp–liquid triple point the Clapeyron slope of the fcc–liquid curve is d*T*/d*P* = 12.8 K/GPa while the slope of the hcp–liquid curve is higher (d*T*/d*P* = 13.7 K/GPa). Therefore, the hcp–liquid curve overlaps the metastable fcc–liquid curve at a pressure of ≈160 GPa. At higher pressures, the fcc phase is in the stability field of the liquid. These transitions can also be confirmed by the temperature dependence of the Gibbs energy of the fcc, hcp, and liquid iron at 125 GPa (Fig. 4). At 125 GPa the fcc-Fe melts at 4019 K, however, this point is in the stability field of the hcp-Fe. The hcp-Fe melts at 4033 K and at higher temperatures the liquid is the only stable phase.

Meanwhile, this assumption is made based on the equilibrium thermodynamics, the shock data^93^ on the iron shows two phase transitions on the Hugoniot curve at pressures from 77 to 400 GPa. A discontinuity in sound velocities of iron at 200 GPa may mark the transition of hcp-iron to fcc iron. The authors described the second discontinuity at 243 GPa as melting of the fcc-Fe. The study^94^ based on new measurements of sound velocity and reassessment of previously obtained shock data established the melting temperature of the iron between 5100 ± 500 K at *P* = 225 ± 3 GPa and 6100 ± 500 K and *P* = 260 ± 3 GPa on the Hugoniot curve. According to the small temperature difference between the melting lines of hcp–liquid and fcc–liquid, there might be a possible fcc–hcp transition in a very narrow temperature range. The following transition can be caused by the kinetics of the transition process or the effect of fcc-Fe and hcp-Fe magnetic moment.

### Magnetism and melting of iron

The magnetic moment of the hcp-Fe is *В*_0_ = 0.28 at ambient conditions^10^. At high pressure and temperature, iron was found to be paramagnetic^47^. However, under the Earth’s core conditions (*P* ≈ 360 GPa, *T* ≈ 6000 K) Fe acquires a substantial local magnetic moment^47^, up to 1.3 μ_B_. The authors used a microscopic phenomenological model for longitudinal spin fluctuations (LSFs) based on density functional theory calculations. In our model (eq. 19) it means that *B*_0_ = 0.3 and this value seems to be too high for the EoS of hcp-Fe. If *B*_0_ = 0.14, then melting of the hcp-Fe is observed at 7150 K and 328.9 GPa and is in agreement with the computer calculations^90^. Whereas the melting curve goes 500 K higher than the experimentally determined one^14^ at 100–200 GPa. The lower value of the hcp-Fe magnetic moment seems to be more realistic. If the magnetic moment for hcp-Fe is *В*_0_ = 0.04 and *T*_N_ = 67 K (see eqs 19,20,21), then the hcp–liquid curve shifts to higher temperatures (red squares on Fig. 3) and crosses the melting curve of fcc-Fe at a temperature of ≈5300 K and pressure of ≈230 GPa. This result is consistent with the data from the shock wave experiments^93^,^94^. The metastable line bcc–hcp-iron was also calculated at high pressures. The extrapolation of this line from the low-pressure data (Fig. 1) is presented in Fig. 3. The bcc–hcp line is located in the stability field of either the fcc-Fe or liquid at high pressures. Thus, the bcc-Fe could not be stable at high pressures based on our thermodynamic model.

### Helmholtz free energy, Gibbs energy, and thermodynamics of iron

Recently, thermodynamic functions for fcc, hcp, and liquid iron were tabulated^20^. It is important to compare the thermodynamics of these phases calculated by different methods. The thermodynamic formalism in ref. 18 and 20 is based on the Gibbs free energy calculations depending on temperature and pressure. First, two partial derivatives of the Gibbs energy allow calculation of the entropy 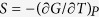 and volume 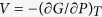. The Helmholtz free energy depends on temperature and volume; thus, its derivatives yield entropy 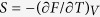 and pressure 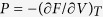. Figures S9–S11 show comparisons of the thermodynamic functions calculated by these two methods for fcc, hcp, and liquid iron. These figures show that the entropy and molar volume of fcc- and hcp-Fe are consistent, especially at the conditions of their stability field. At the same time, our EoSs cannot be extrapolated to very high temperature at 0.1 MPa. At moderate and high pressures, such extrapolation provides reliable results with a close agreement with reference data^20^. At 0.1 MPa the calculated entropy^20^ of solid phases of Fe decreases much faster than in our EoSs. The volumes for liquid iron from our EoS and that from ref. 20 are very consistent; however, entropy is consistent only at temperatures close to the melting line (Supplementary Figure S11).

Supplementary Figure S12 shows isochores of liquid Fe calculated from our EoS and plotted in comparison with *P–V–T* data calculated by *ab initio* molecular dynamics simulations^95^,^96^. The comparison with earlier *ab initio* calculations can be found in the cited papers.

Comparisons of other thermodynamic functions, which are second derivatives of the Gibbs energy or the Helmholtz free energy, are not necessary. The values of these functions can be found in Tables S1–S4. If entropy and molar volume (pressure) are comparable in different EoSs, then the Gibbs energy will be similar and deviations in calculated lines in equilibrium phase diagram would be negligible. The differences would be defined by reference points chosen by different authors.

### Comparison of properties of the Earth’s core from PREM with iron

**Figure 5:**
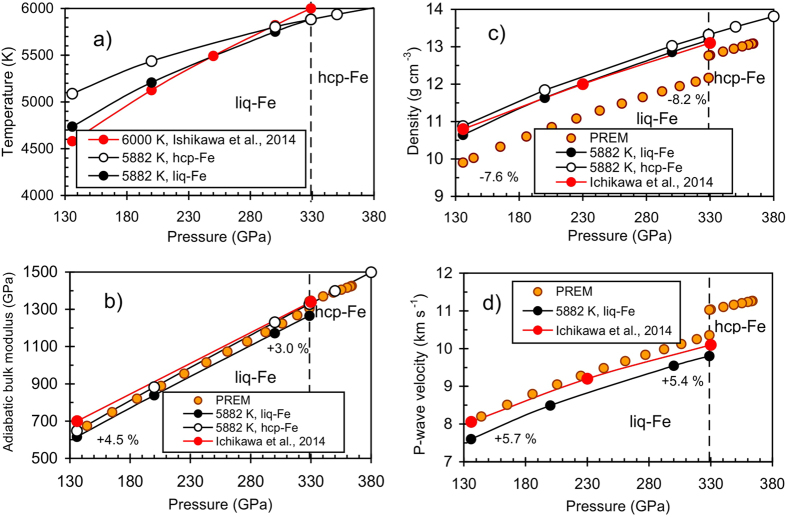
Comparison of the physical properties of liquid and hcp-iron calculated along an isentropic temperature profile (*T*_ICB_ = 5882 K) with the PREM^72^ and reference data^95^ (*T*_ICB_ = 6000 K). (**a**) Calculated isentropic temperature profile started from 5882 K at 328.9 GPa for liquid and hcp-iron compared with the reference data^95^ (**b**) Calculated adiabatic bulk modulus along adiabats for solid and liquid iron. (**c**) Calculated density along the 5882 K isentrope. (**d**) Calculated P-wave velocity along the isentrope.

Density (*ρ* at given *P* and *T*), adiabatic bulk modulus (*K*_*S*_) and the P-wave velocity (*v*_P_ = (*K*_*S*_/ρ)^1/2^) of the liquid iron estimated by our formalism can be compared with the values of PREM^72^. The following comparison has also been assessed by previous studies^84^,^95^ on temperature calculations of liquid iron in the Earth’s core at isentropic conditions and various pressures. The initial temperature in our model is 5882 K at the inner core boundary (ICB) (328.9 GPa) (Fig. 3). At these conditions, the entropy for liquid iron, *S* = 114.15 J mol^−1 ^K^−1^ and hcp-Fe, *S* = 107.2 J mol^−1 ^K^−1^, were determined from eqs 7 and 17. Under isentropic conditions, the entropy at the pressures of the mantle–core boundary (MCB, 135.8 GPa) is equal to the entropy at the ICB. Figure 5a shows the calculated isentropic temperature profile started from ICB conditions for liquid iron. The calculated isentropic temperature profile for hcp-iron and liquid iron started from 6000 K based on the reference data^95^ are also presented in Fig. 5a. The Clapeyron slope (∂*T*/∂*P*)_*S*_ for hcp-Fe differs significantly for both the liquid Fe and the calculated model^95^. This inconsistency is probably due to the differences in the EoSs.

Figure 5b shows adiabatic bulk modulus and density for solid and liquid iron calculated from our EoSs in comparison with PREM^72^ and the calculated model^95^. The deviations from the PREM are presented as (*K*_*S* PREM_ − *K*_*S* cal_)/*K*_*S* PREM_) × 100. The adiabatic bulk modulus for liquid Fe is about 3.0–4.5% lower than the PREM and the calculated^95^ *K*_*S*_ is 3.1–9.0% higher than the PREM (Fig. 5b). *K*_*S*_ for hcp-Fe is almost identical to the PREM at the Earth’s inner core conditions. The calculated isentrope for liquid iron from 5000 K to 8000 K is presented in ref. 84. Based on these data, the adiabatic bulk modulus at the ICB conditions is consistent with isentropes of 7000 K and at the ICB with isentropes of 5000 K (see Fig. 10 in ref. 84). The liquid iron density calculated in our study is 7.6–8.2% higher than PREM and the calculated one^95^ is 8.9–7.7% higher than PREM (Fig. 5c). Figure 5d shows that the calculated P-wave velocity for liquid iron is 5.4–5.7% lower than PREM and consistent with the calculated estimations^84^. The calculations^95^ indicate that the P-wave velocity is 2.5% lower than PREM at the ICB conditions and very close to PREM at the CMB.

## Summary

The EoSs of the liquid and solid iron phases were constrained based on the Helmholtz free energy. The obtained EoSs allow calculation of *P–V–T* relations and thermodynamic properties of Fe at the Earth’s core conditions (up to 6000 K and 350 GPa). The calculated triple points have the following parameters: bcc–fcc–hcp is located at 7.3 GPa and 820 K, bcc–fcc–liquid at 5.2 GPa and 1998 K, and fcc–hcp–liquid at 106.5 GPa and 3787 K. The melting temperature is 5882 К at ICB pressure (328.9 GPa). An extrapolation to high-pressure conditions revealed the very close position of the melting curves of fcc- and hcp-Fe. If the magnetic moment for hcp-Fe is *В*_0_ = 0.04 and *T*_N_ = 67 K (see eqs 19,20,21), the hcp–liquid curve shifts to higher temperatures and crosses the fcc–liquid curve at the temperature of ≈5300 K and pressure of ≈230 GPa. This estimation is consistent with the shock wave data^93^,^94^. At higher pressure conditions, the metastable bcc–hcp curve is in the fcc-Fe or liquid stability field. The iron density, adiabatic bulk modulus and P-wave velocity calculated up to 328.9 GPa at adiabatic temperature conditions started from 5882 K (outer/inner core boundary) were compared with the PREM and calculated model^95^. We determined the density deficit of hcp-Fe at the inner core boundary (*T* = 5882 K and *P* = 328.9 GPa) to be 4.4%.

## Additional Information

**How to cite this article**: Dorogokupets, P. I. *et al*. Thermodynamics and Equations of State of Iron to 350 GPa and 6000 K. *Sci. Rep.* **7**, 41863; doi: 10.1038/srep41863 (2017).

**Publisher's note:** Springer Nature remains neutral with regard to jurisdictional claims in published maps and institutional affiliations.

## Supplementary Material

Supplementary Figures

Supplementary Dataset 1

Supplementary Dataset 2

Supplementary Dataset 3

Supplementary Dataset 4

## Figures and Tables

**Table 1:** Fitting parameters of EoSs for solid and liquid phases of iron.

Parameter	bcc-Fe α	fcc-Fe γ	hcp-Fe ε	Liquid Fe *T*_0_ = 1811 K
*U*_0_ (kJ mol^−1^)	0	4.470	4.500	−100.204
*V*_0_ (cm^3 ^mol^−1^)	7.092	6.9285	6.8175	7.957^84^
*K*_0_ (GPa)	164.0	146.2	148.0	83.7
*K*_0_′	5.50	4.67	5.86	5.97
Θ_0_ (K)	303	222.5	227	263
*γ*_0_	1.736	2.203	2.20	2.033
β	1.125	0.01	0.01	1.168
*γ*_∞_	0	0	0	0
*e*_0_ (10^−6 ^K^−1^)	198	198	126	198
*g*	1.0	0.5	−0.83	0.884
*T*_C_ or *T*_N_ (K)	1043			
*B*_0_	2.22			
*a*_*S*_				2.12
