# Supplementary material for: Thermodynamics and Equations of State of Iron to 350 GPa and 6000 K
Source: Sci Rep. 2017 Mar 6;7:41863. doi: 10.1038/srep41863 (PMC5338021; doi:10.1038/srep41863)
Supplement: Supplementary Figures [file srep41863-s1.doc]

## Supplementary material

**Thermodynamics and Equations of State of Iron to 350 GPa and 6000 K**

**P. I. Dorogokupets ^1^, A.M. Dymshits ^2^**, **K. D. Litasov ^2,3^**, **T. S. Sokolova ^1^**

*^1^ Institute of Earth’s Crust, SB RAS, Irkutsk, Russia*

*^2^ V.S. Sobolev Institute of Geology and Mineralogy SB RAS, Novosibirsk, Russia*

*^3^ Novosibirsk State University, Novosibirsk, Russia*

*e-mail: dor@crust.irk.ru*

Contents of this file

Figures S1 to S12.

Tables S1 to S4.


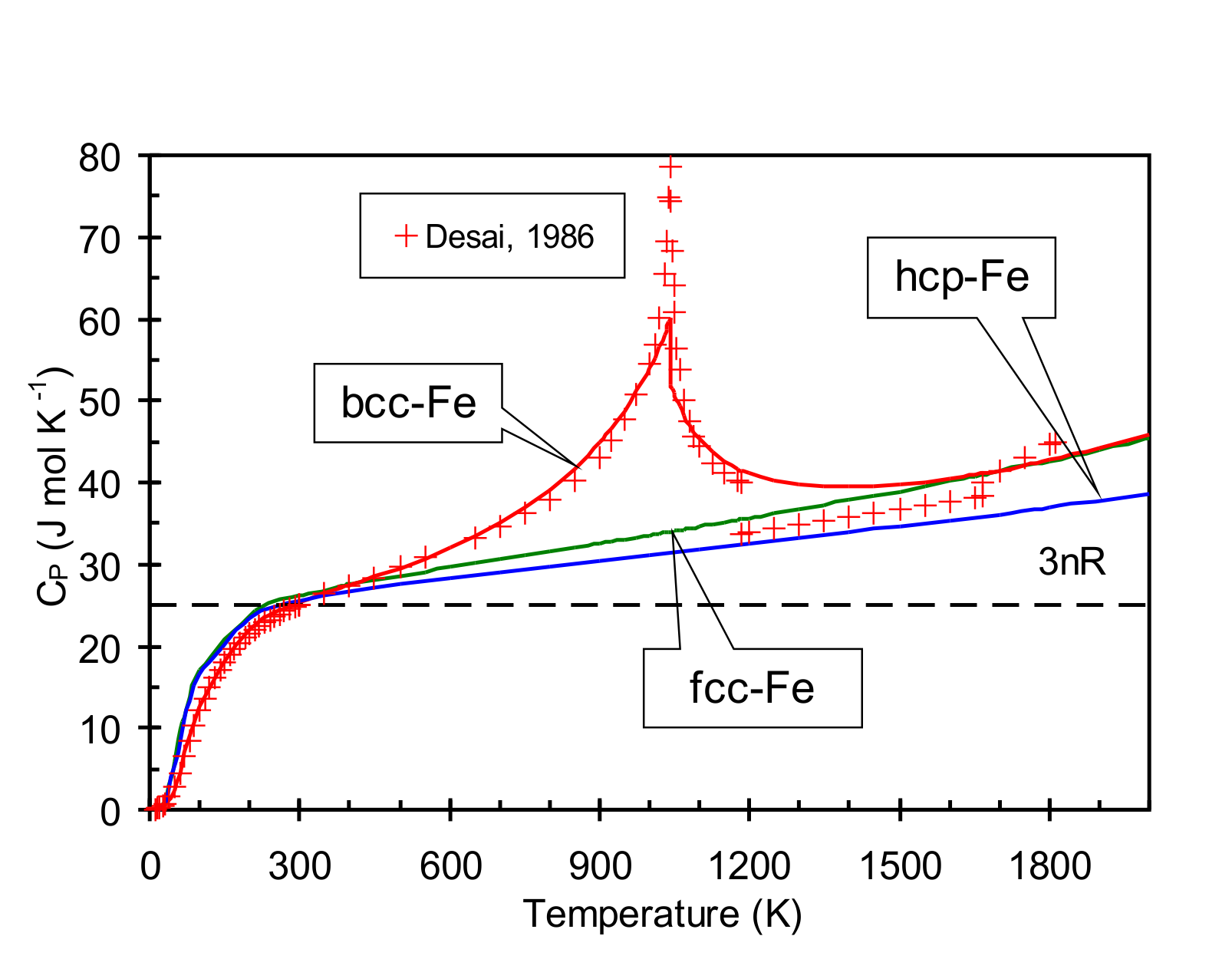


Figure S1. Calculated heat capacity of bcc-Fe, fcc-Fe, and hcp-Fe at 0.1 MPa in comparison with experimental data by .

#
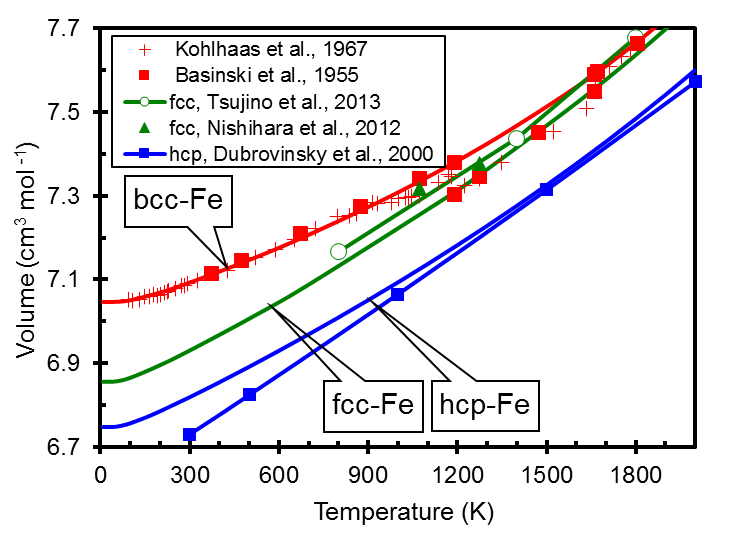


Figure S2. Calculated volumes of bcc-Fe, fcc-Fe and hcp-Fe at 0.1 MPa in comparison with those from measurements and and estimations from high-pressure experiments .


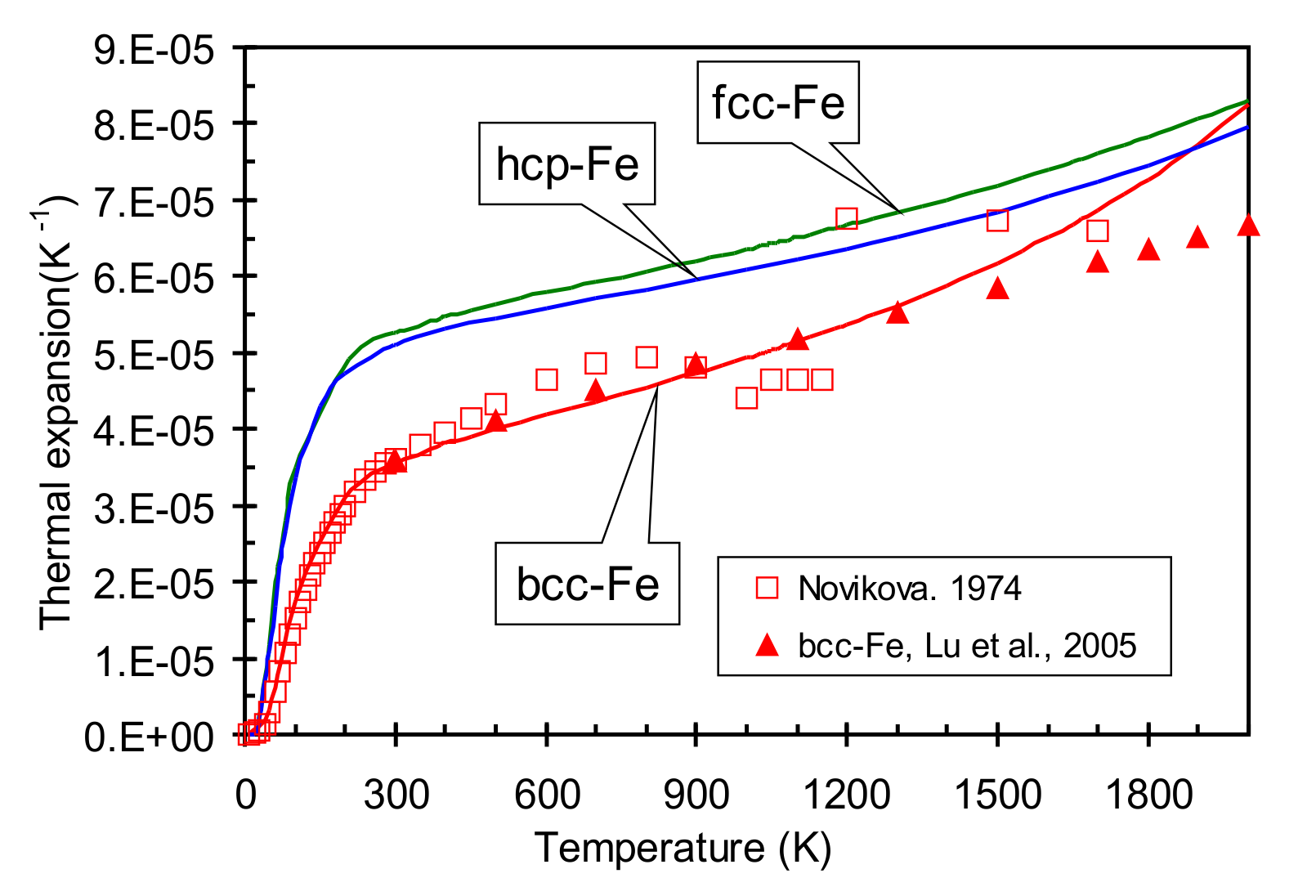


Figure S3. Calculated thermal expansion coefficient of bcc-Fe, fcc-Fe and hcp-Fe at 0.1 MPa in comparison with reference data .


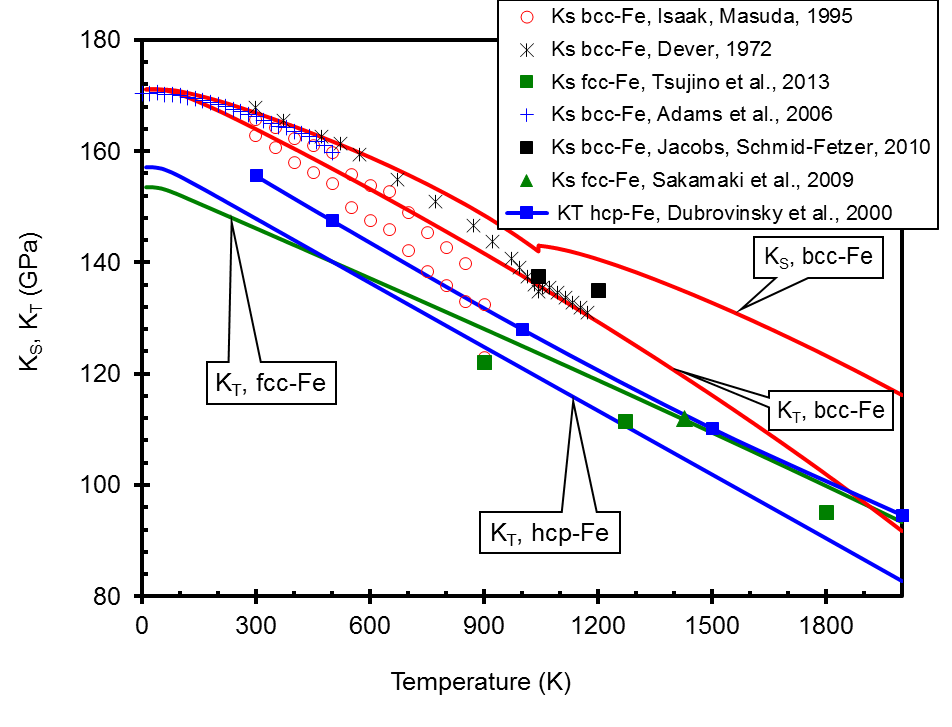


Figure S4. Calculated bulk moduli of bcc-Fe, fcc-Fe and hcp-Fe at 0.1 MPa in comparison with measured *K_S_* and *K_T_* for hcp-Fe .


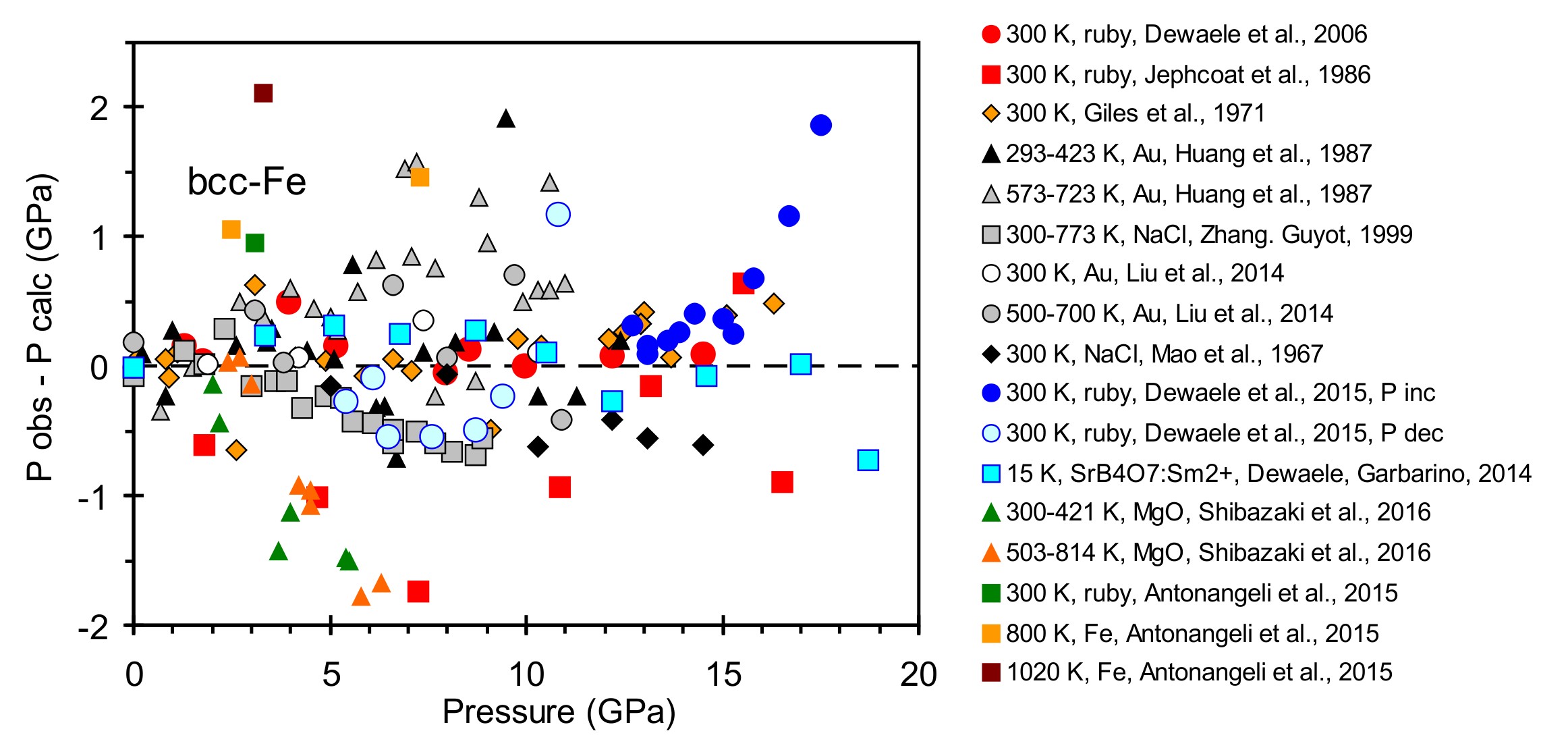


Figure S5. The differences between measured pressures and calculated pressures from the present EoS for bcc-Fe. Pressure marker and temperature are noticed in the legend.


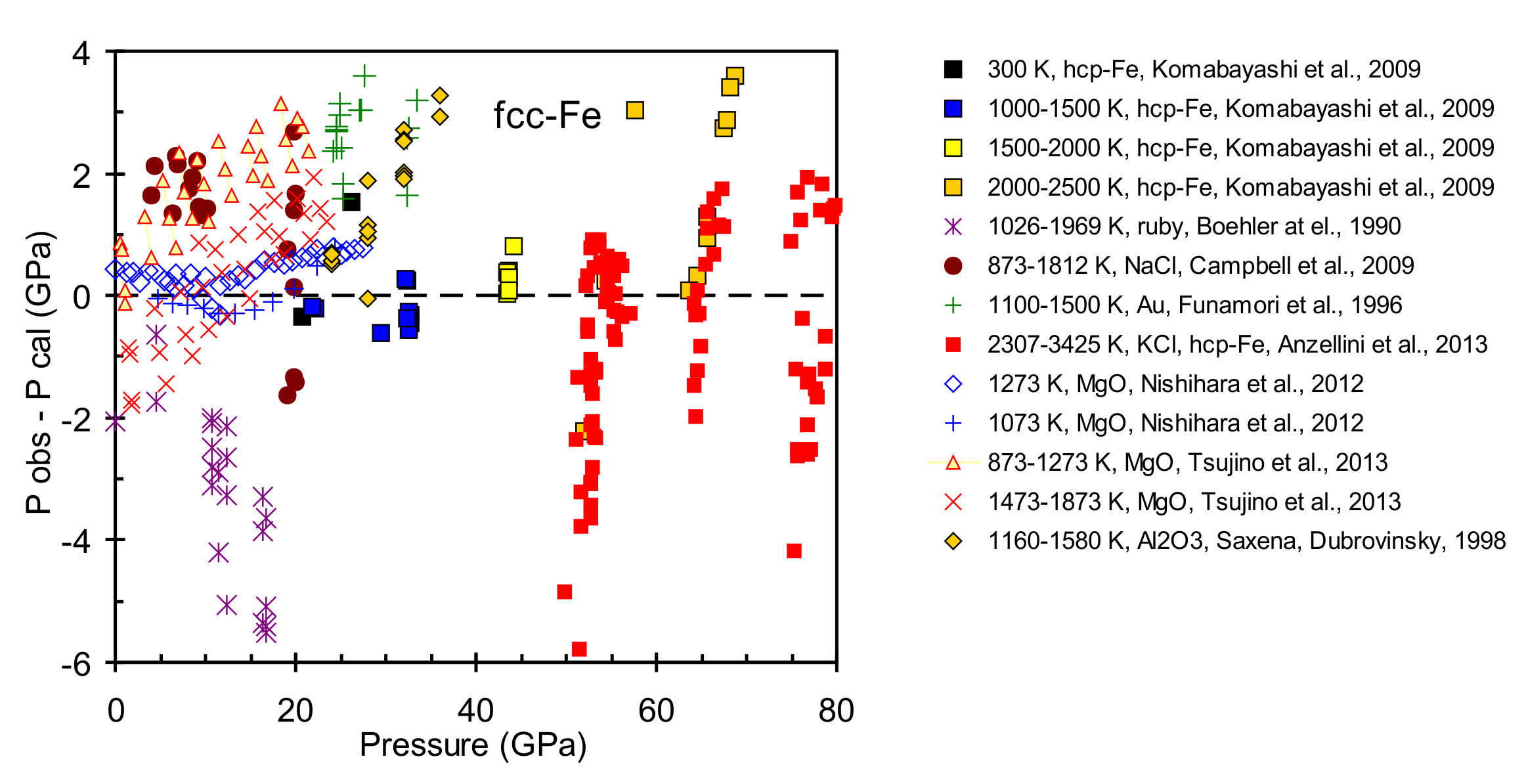


Figure S6. Difference between measured pressures and calculated pressures from the present EoS for fcc-Fe. Pressure markers and temperature are noticed in the legend.


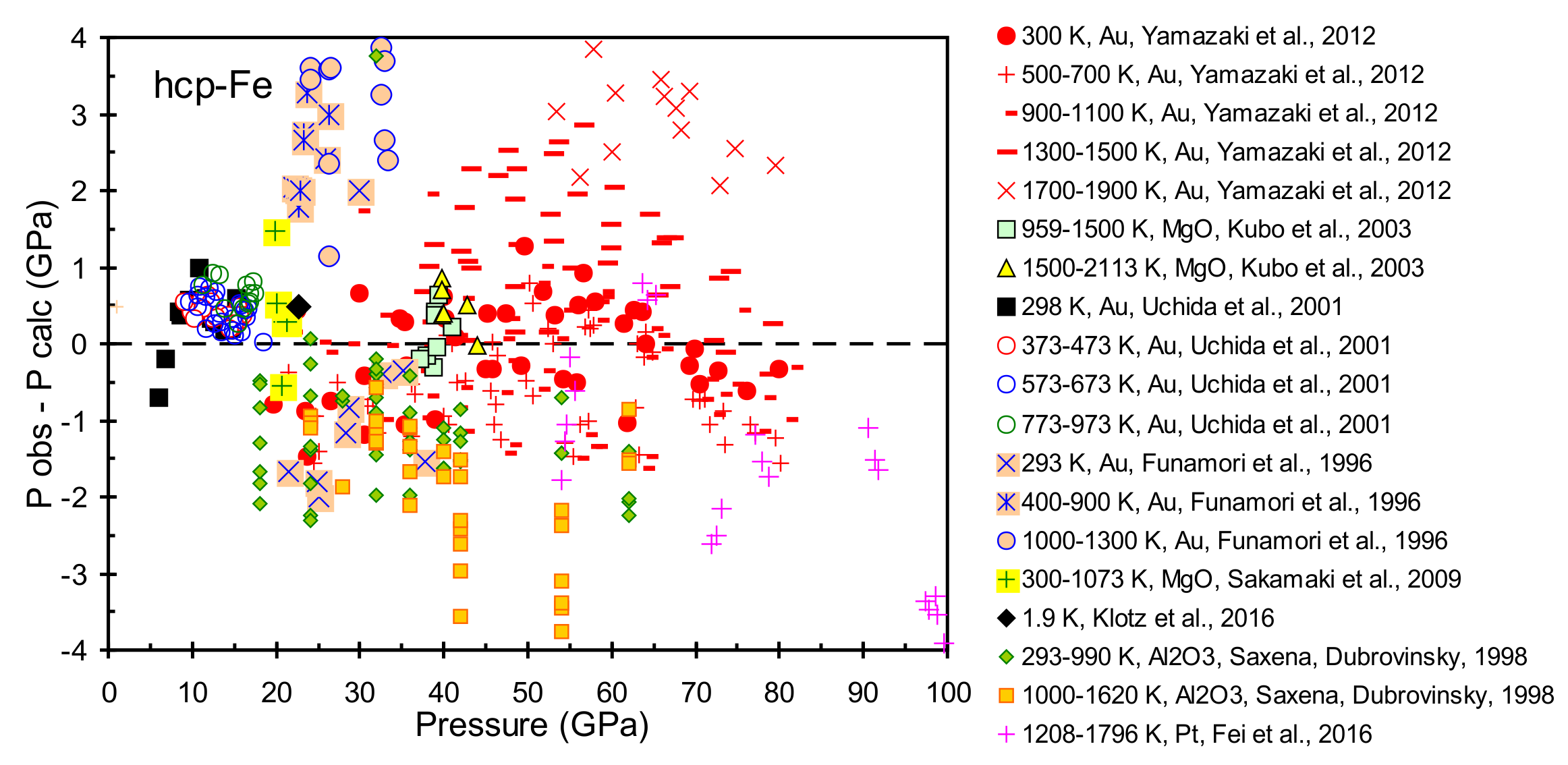


Figure S7. Difference between measured pressures and calculated pressures from the present EoS for hcp-Fe in the pressure range up to 80 GPa. Pressure markers and temperature are noticed in the legend.


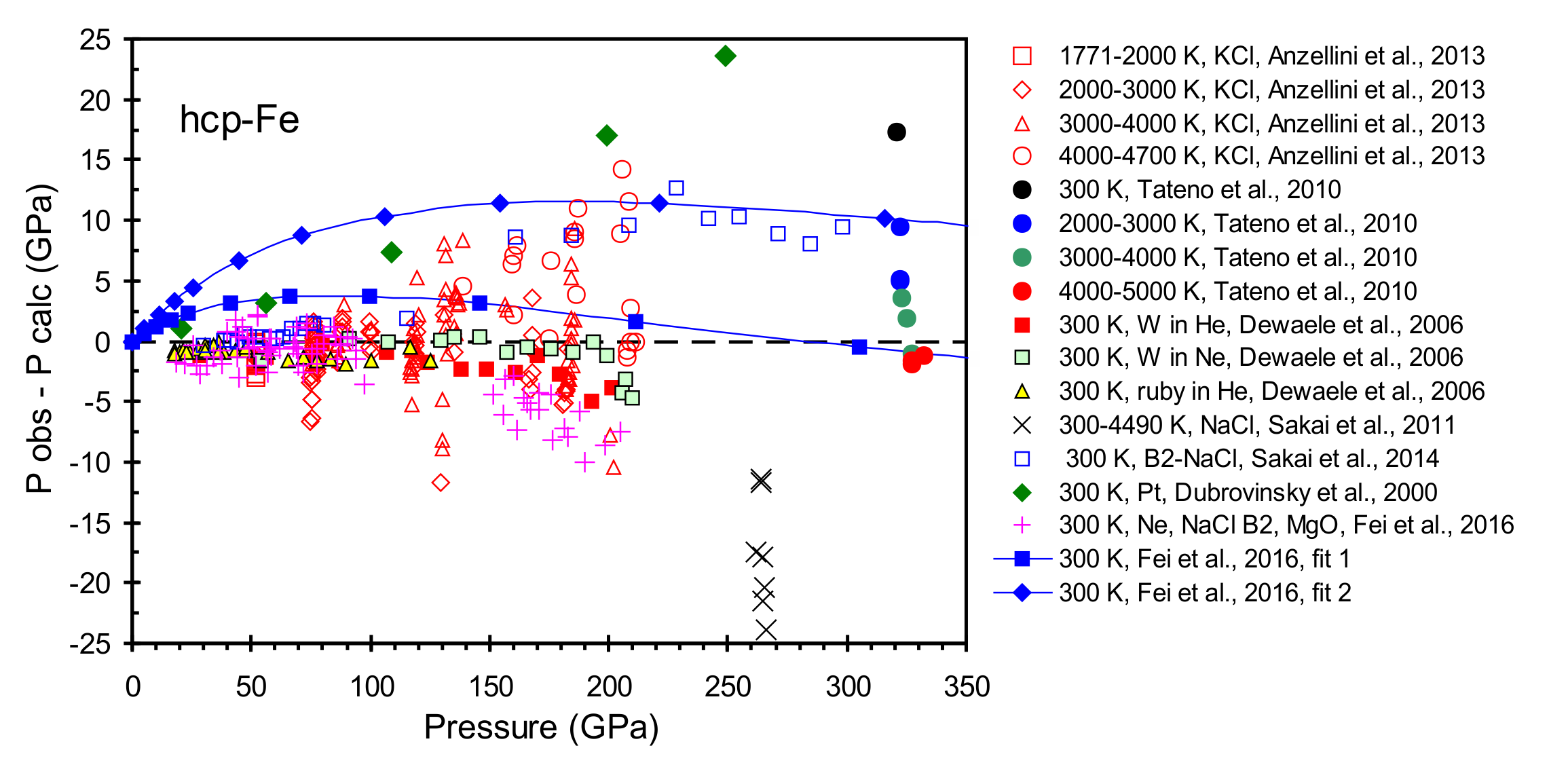


Figure S8. Difference between measured pressures and calculated pressures from the present EoS for hcp-Fe in the pressure range up to ≈ 340 GPa. Pressure marker and temperatures are noticed in the legend. Fei et al. (2016) fits with a third-order Birch-Murnaghan equation of state: fit 1 with fixed ρ_0_ = 8.2695 g cm^-3^, *K*_0_ = 172.7 GPa, *K*_0_*'* = 4.79; fit 2 (without constraining ρ_0_) ρ_0_ = 8.3602 g cm^-3^, *K*_0_ = 191.44 GPa, *K*_0_*'* = 4.52.


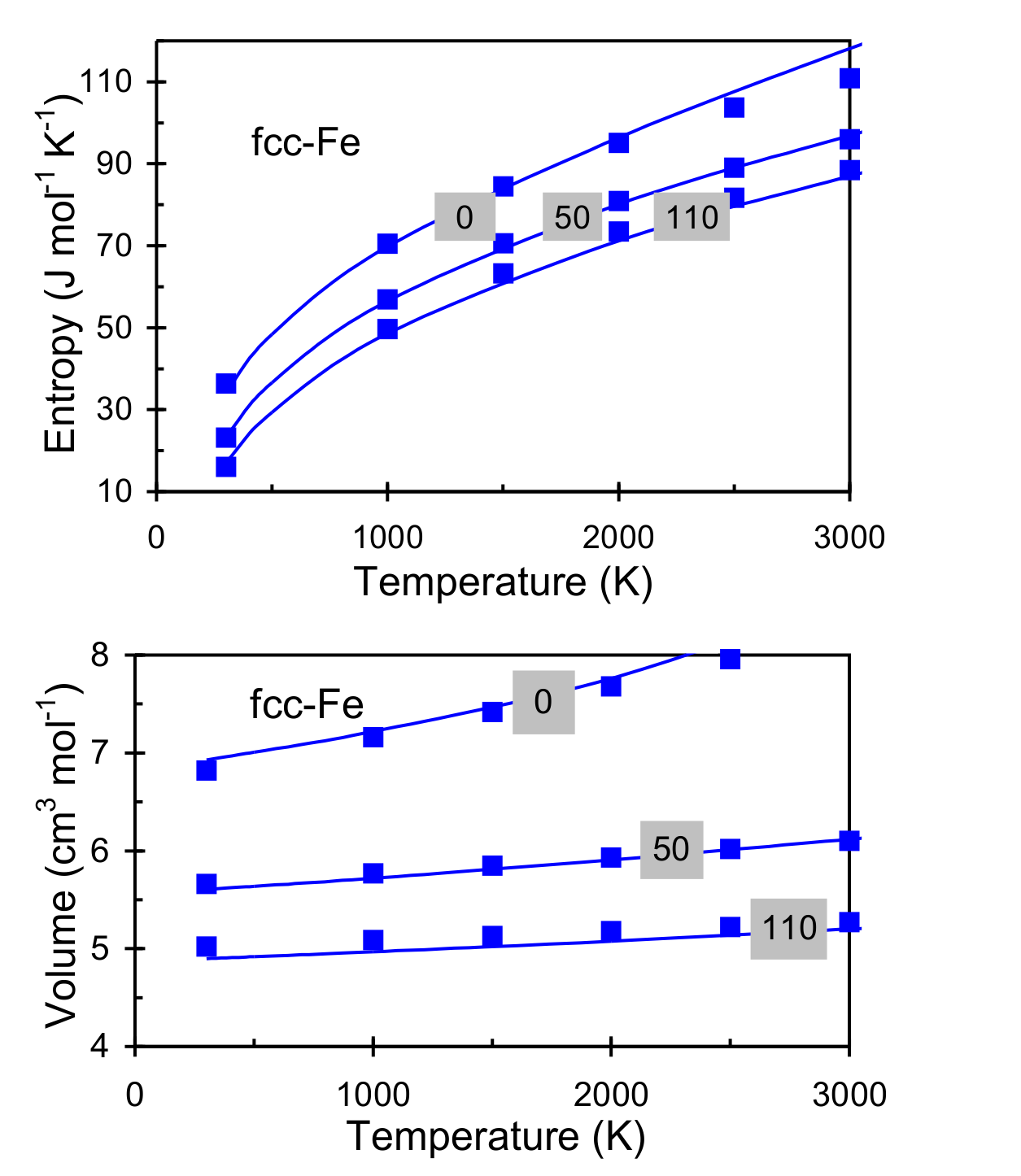


Figure S9. Calculated (lines) entropy and molar volume of fcc-Fe in comparison with data from (squares) on 0.0001, 50 and 110 GPa isobars.


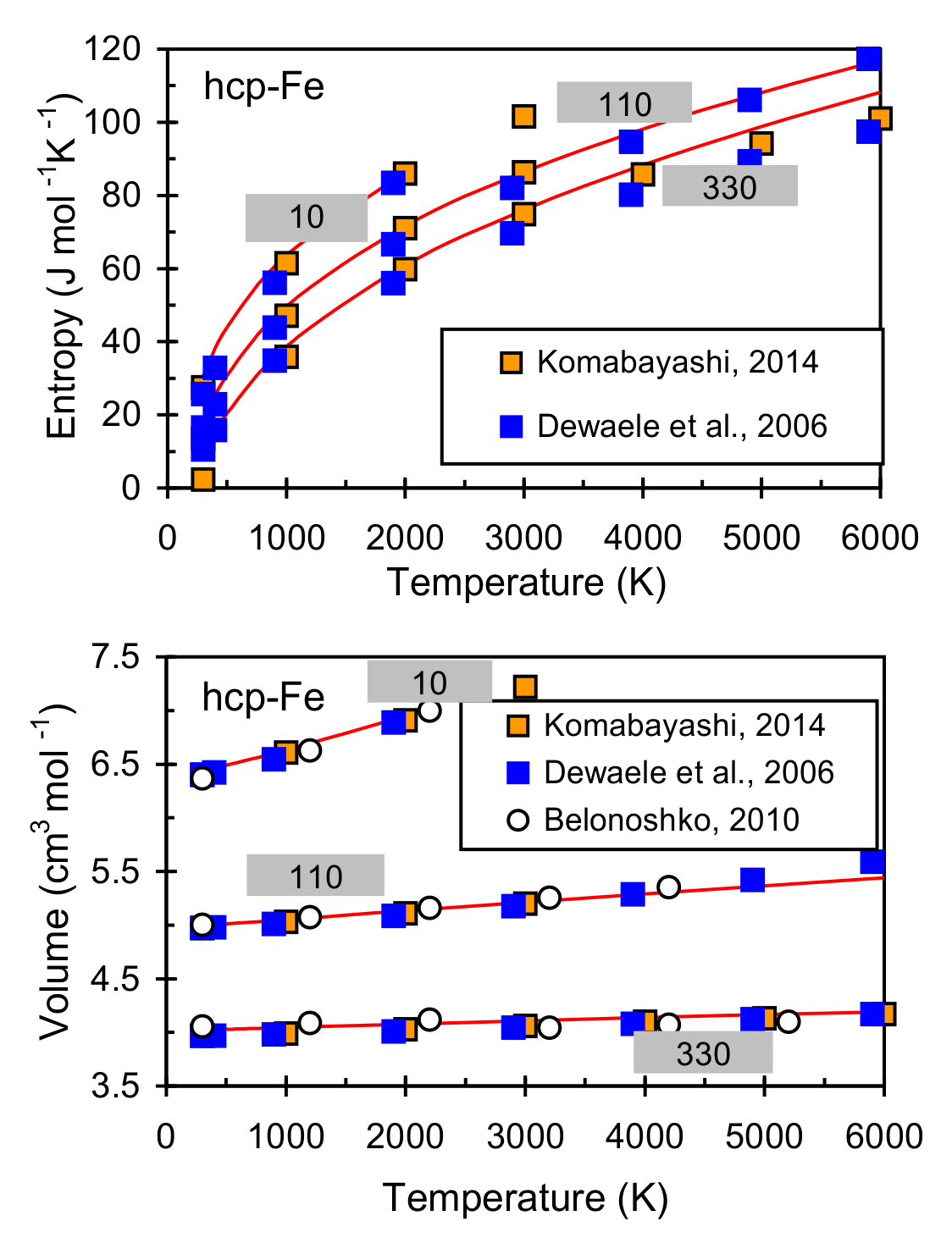


Figure S10. Calculated (lines) entropy and molar volume of hcp-Fe in comparison with data from on 10, 110 and 330 GPa isobars.


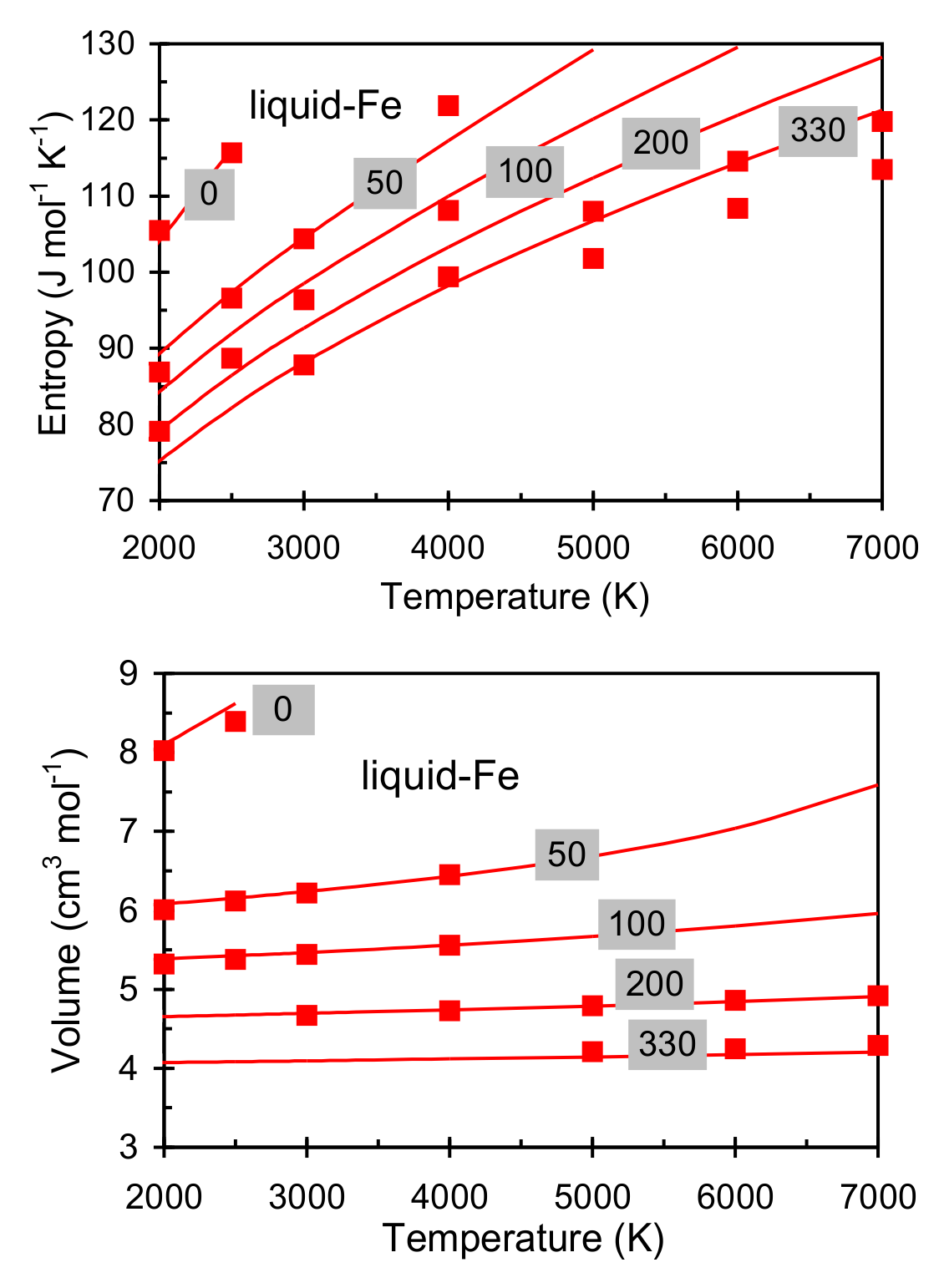


Figure S11. Calculated (lines) entropy and molar volume of liquid iron in comparison with data from (squares) on 0.1 MPa, 50, 100, 200 and 330 GPa isobars.


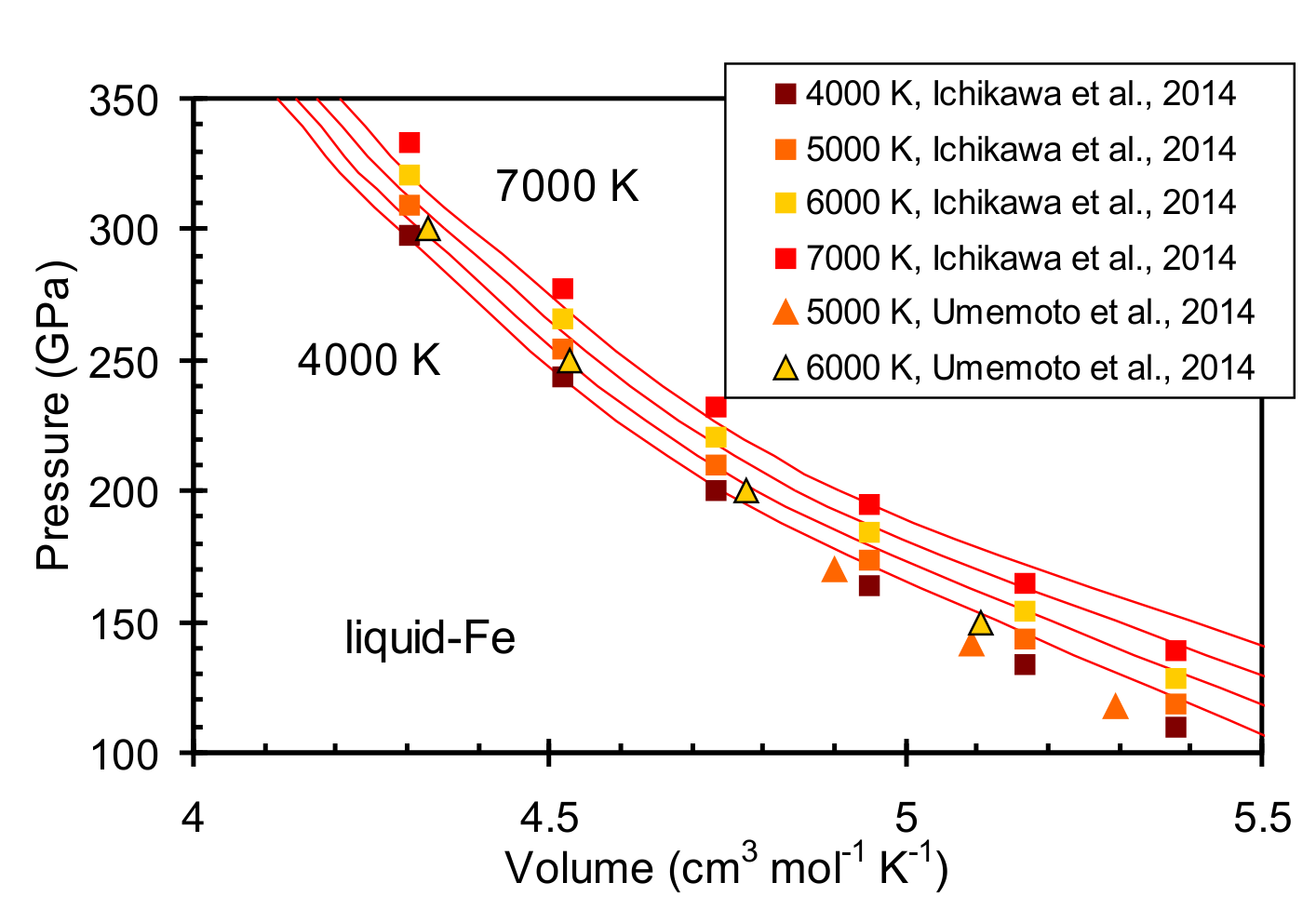


Figure S12. Calculated (lines) molar volume of liquid iron along 4000, 5000, 6000 and 7000 K isotherms in comparison with data from (squares) and (triangles) along 5000 and 6000 K isotherms.

Table S1. Thermodynamic properties of bcc-Fe.

| P, GPa | T, K | x=V/V_0_ | αE–6,  K^-1^ | *S* | *C_V_* | *C_P_* | *K_T_* | *K_S_* | γ_th_ | *K'* | *G*,  kJ mol^-1^ |
| --- | --- | --- | --- | --- | --- | --- | --- | --- | --- | --- | --- |
|  |  |  |  | J mol^-1^K^-1^ | | | GPa | |  |  |  |
| 0.0001 | 298.15 | 1.00000 | 35.45 | 27.199 | 24.793 | 25.229 | 164.001 | 166.883 | 1.736 | 5.50 | 0.001 |
| 0.0001 | 500 | 1.00769 | 40.05 | 41.283 | 28.677 | 29.575 | 156.840 | 161.755 | 1.751 | 5.58 | -6.996 |
| 0.0001 | 1043 | 1.03260 | 50.20 | 69.439 | 57.480 | 60.094 | 135.830 | 142.007 | 1.800 | 5.85 | -37.130 |
| 0.0001 | 1185 | 1.04022 | 53.36 | 75.213 | 38.221 | 41.455 | 129.918 | 140.909 | 1.815 | 5.93 | -47.424 |
| 0.0001 | 1500 | 1.05920 | 61.82 | 84.610 | 34.768 | 39.768 | 116.094 | 132.790 | 1.852 | 6.17 | -72.663 |
| 0.0001 | 1667 | 1.07068 | 67.45 | 88.872 | 34.905 | 41.142 | 108.317 | 127.672 | 1.875 | 6.33 | -87.153 |
| 0.0001 | 1811 | 1.08156 | 73.20 | 92.350 | 35.365 | 42.906 | 101.321 | 122.925 | 1.896 | 6.49 | -100.203 |
| 0.0001 | 2000 | 1.09755 | 82.49 | 96.750 | 36.232 | 45.943 | 91.669 | 116.236 | 1.928 | 6.74 | -118.075 |
| 10 | 298.15 | 0.94858 | 26.26 | 25.102 | 24.343 | 24.643 | 216.541 | 219.205 | 1.636 | 5.05 | 68.996 |
| 10 | 500 | 0.95397 | 29.54 | 38.905 | 28.398 | 29.018 | 210.035 | 214.621 | 1.646 | 5.09 | 62.454 |
| 10 | 1000 | 0.96948 | 34.91 | 64.126 | 51.227 | 52.842 | 192.708 | 198.782 | 1.677 | 5.22 | 36.576 |
| 10 | 1500 | 0.98799 | 40.96 | 81.000 | 34.221 | 37.286 | 173.817 | 189.385 | 1.713 | 5.39 | -0.269 |
| 10 | 1811 | 1.00136 | 45.62 | 88.149 | 34.634 | 38.948 | 161.203 | 181.285 | 1.739 | 5.51 | -26.598 |
| 10 | 2000 | 1.01034 | 48.93 | 92.094 | 35.359 | 40.615 | 153.177 | 175.945 | 1.756 | 5.60 | -43.635 |
| 15 | 298.15 | 0.92807 | 23.33 | 24.279 | 24.148 | 24.405 | 241.387 | 243.964 | 1.596 | 4.89 | 102.262 |
| 15 | 500 | 0.93277 | 26.25 | 37.974 | 28.280 | 28.817 | 235.107 | 239.563 | 1.605 | 4.93 | 95.898 |
| 15 | 1000 | 0.94617 | 30.70 | 63.015 | 51.094 | 52.475 | 218.416 | 224.319 | 1.631 | 5.03 | 70.530 |
| 15 | 1500 | 0.96190 | 35.43 | 79.685 | 34.019 | 36.593 | 200.317 | 215.468 | 1.662 | 5.16 | 34.290 |
| 15 | 1811 | 0.97308 | 38.93 | 86.676 | 34.375 | 37.941 | 188.307 | 207.844 | 1.684 | 5.25 | 8.393 |
| 15 | 2000 | 0.98048 | 41.34 | 90.509 | 35.059 | 39.355 | 180.701 | 202.843 | 1.698 | 5.32 | -8.355 |

Table S2. Thermodynamic properties of fcc-Fe.

| *P*, GPa | *T*, K | *x=V/V*_0_ | αE–6,  K^-1^ | *S* | *C_V_* | *C_P_* | *K_T_* | *K_S_* | γ_th_ | *K*' | *G*,  kJ mol^-1^ |
| --- | --- | --- | --- | --- | --- | --- | --- | --- | --- | --- | --- |
|  |  |  |  | J mol^-1^K^-1^ | | | GPa | |  |  |  |
| 0.0001 | 298.15 | 1.00000 | 52.53 | 34.287 | 25.290 | 26.123 | 146.200 | 151.017 | 2.104 | 4.67 | 4.471 |
| 0.0001 | 500 | 1.01109 | 56.36 | 48.424 | 27.038 | 28.598 | 140.164 | 148.249 | 2.047 | 4.75 | -3.978 |
| 0.0001 | 1043 | 1.04471 | 64.17 | 71.187 | 30.131 | 33.975 | 123.664 | 139.441 | 1.906 | 5.02 | -37.027 |
| 0.0001 | 1185 | 1.05443 | 66.40 | 75.615 | 30.895 | 35.448 | 119.270 | 136.845 | 1.873 | 5.11 | -47.455 |
| 0.0001 | 1500 | 1.07764 | 71.95 | 84.360 | 32.601 | 38.943 | 109.390 | 130.671 | 1.803 | 5.33 | -72.683 |
| 0.0001 | 1667 | 1.09096 | 75.31 | 88.574 | 33.518 | 40.955 | 104.072 | 127.165 | 1.768 | 5.47 | -87.126 |
| 0.0001 | 1811 | 1.10311 | 78.50 | 92.042 | 34.317 | 42.798 | 99.437 | 124.011 | 1.738 | 5.61 | -100.132 |
| 0.0001 | 2000 | 1.12008 | 83.17 | 96.415 | 35.382 | 45.396 | 93.280 | 119.682 | 1.702 | 5.81 | -117.944 |
| 10 | 298.15 | 0.94216 | 42.09 | 31.139 | 24.921 | 25.579 | 190.838 | 195.877 | 2.104 | 4.29 | 71.643 |
| 10 | 500 | 0.95053 | 45.13 | 45.016 | 26.843 | 28.084 | 185.071 | 193.630 | 2.049 | 4.34 | 63.858 |
| 10 | 1000 | 0.97335 | 49.61 | 65.875 | 29.700 | 32.534 | 170.712 | 187.000 | 1.923 | 4.49 | 35.664 |
| 10 | 1500 | 0.99891 | 54.19 | 79.910 | 32.302 | 37.061 | 156.118 | 179.120 | 1.813 | 4.66 | -0.943 |
| 10 | 2000 | 1.02769 | 59.58 | 91.245 | 34.934 | 42.074 | 141.253 | 170.123 | 1.715 | 4.88 | -43.807 |
| 10 | 2500 | 1.06045 | 66.18 | 101.232 | 37.646 | 47.789 | 126.097 | 160.075 | 1.629 | 5.17 | -91.966 |
| 50 | 298.15 | 0.80938 | 25.42 | 23.378 | 23.528 | 23.903 | 347.028 | 352.559 | 2.103 | 3.64 | 312.077 |
| 50 | 500 | 0.81379 | 27.92 | 36.560 | 26.176 | 26.927 | 341.512 | 351.304 | 2.054 | 3.66 | 305.939 |
| 50 | 1000 | 0.82574 | 30.14 | 56.525 | 29.194 | 30.899 | 328.084 | 347.242 | 1.938 | 3.71 | 282.209 |
| 50 | 1500 | 0.83862 | 31.74 | 69.714 | 31.629 | 34.392 | 314.605 | 342.089 | 1.835 | 3.76 | 250.485 |
| 50 | 2000 | 0.85238 | 33.36 | 80.095 | 34.011 | 37.969 | 300.997 | 336.021 | 1.744 | 3.82 | 212.952 |
| 50 | 2500 | 0.86709 | 35.09 | 88.966 | 36.410 | 41.722 | 287.249 | 329.157 | 1.663 | 3.89 | 170.639 |
| 50 | 3000 | 0.88284 | 36.96 | 96.921 | 38.845 | 45.697 | 273.367 | 321.587 | 1.591 | 3.97 | 124.137 |
| 100 | 298.15 | 0.72032 | 17.66 | 17.811 | 21.820 | 22.061 | 519.621 | 525.363 | 2.099 | 3.30 | 575.709 |
| 100 | 500 | 0.72312 | 20.27 | 30.294 | 25.399 | 25.928 | 513.994 | 524.705 | 2.056 | 3.31 | 570.779 |
| 100 | 1000 | 0.73089 | 22.09 | 49.655 | 28.761 | 29.998 | 500.644 | 522.177 | 1.947 | 3.34 | 550.352 |
| 100 | 1500 | 0.73919 | 23.07 | 62.418 | 31.141 | 33.134 | 487.383 | 518.578 | 1.849 | 3.37 | 522.171 |
| 100 | 2000 | 0.74793 | 23.96 | 72.371 | 33.394 | 36.215 | 474.032 | 514.073 | 1.763 | 3.40 | 488.392 |
| 100 | 2500 | 0.75712 | 24.85 | 80.788 | 35.631 | 39.362 | 460.560 | 508.787 | 1.685 | 3.44 | 450.053 |
| 100 | 3000 | 0.76676 | 25.78 | 88.251 | 37.881 | 42.615 | 446.960 | 502.816 | 1.616 | 3.47 | 407.761 |
| 100 | 3500 | 0.77689 | 26.75 | 95.072 | 40.154 | 45.993 | 433.236 | 496.236 | 1.553 | 3.51 | 361.909 |
| 100 | 4000 | 0.78755 | 27.77 | 101.441 | 42.457 | 49.515 | 419.392 | 489.111 | 1.497 | 3.55 | 312.764 |

Table S3. Thermodynamic properties of hcp-Fe.

| *P*, GPa | *T*, K | *x=V/V*_0_ | αE–6,  K^-1^ | *S* | *C_V_* | *C_P_* | *K_T_* | *K_S_* | γ_th_ | *K*' | *G*,  kJ mol^-1^ |
| --- | --- | --- | --- | --- | --- | --- | --- | --- | --- | --- | --- |
|  |  |  |  | J mol^-1^K^-1^ | | | GPa | |  |  |  |
| 0.0001 | 298.15 | 1.00000 | 51.06 | 33.275 | 24.710 | 25.494 | 148.001 | 152.699 | 2.085 | 5.86 | 4.501 |
| 0.0001 | 500 | 1.01075 | 54.54 | 46.988 | 26.096 | 27.533 | 140.226 | 147.948 | 2.019 | 5.98 | -3.701 |
| 0.0001 | 1000 | 1.04030 | 60.77 | 67.185 | 27.895 | 31.066 | 121.054 | 134.814 | 1.870 | 6.35 | -32.723 |
| 0.0001 | 1500 | 1.07436 | 68.49 | 80.440 | 29.351 | 34.606 | 101.966 | 120.222 | 1.743 | 6.87 | -69.793 |
| 0.0001 | 2000 | 1.11468 | 79.67 | 90.933 | 30.671 | 38.659 | 82.805 | 104.369 | 1.634 | 7.64 | -112.713 |
| 0.0001 | 2500 | 1.16479 | 97.90 | 100.090 | 31.858 | 43.897 | 63.281 | 87.197 | 1.544 | 9.02 | -160.506 |
| 10 | 298.15 | 0.94439 | 38.71 | 30.349 | 24.433 | 25.018 | 203.629 | 208.510 | 2.077 | 5.32 | 70.661 |
| 10 | 500 | 0.95206 | 41.07 | 43.846 | 26.055 | 27.130 | 196.307 | 204.405 | 2.009 | 5.39 | 63.074 |
| 10 | 1000 | 0.97254 | 43.89 | 63.679 | 28.039 | 30.318 | 178.443 | 192.946 | 1.852 | 5.58 | 35.715 |
| 10 | 1500 | 0.99477 | 46.57 | 76.515 | 29.630 | 33.181 | 160.952 | 180.243 | 1.716 | 5.80 | 0.498 |
| 10 | 2000 | 1.01899 | 49.75 | 86.453 | 31.107 | 36.052 | 143.800 | 166.656 | 1.598 | 6.08 | -40.328 |
| 10 | 3000 | 1.07539 | 58.75 | 102.204 | 33.812 | 42.188 | 110.355 | 137.694 | 1.406 | 6.88 | -134.982 |
| 100 | 298.15 | 0.74476 | 14.54 | 18.882 | 22.171 | 22.366 | 609.879 | 615.246 | 2.030 | 4.09 | 575.656 |
| 100 | 500 | 0.74711 | 16.21 | 31.424 | 25.458 | 25.862 | 603.051 | 612.611 | 1.956 | 4.10 | 570.500 |
| 100 | 1000 | 0.75335 | 16.79 | 50.570 | 28.551 | 29.401 | 586.822 | 604.292 | 1.773 | 4.13 | 549.553 |
| 100 | 1500 | 0.75969 | 16.72 | 62.984 | 30.707 | 31.948 | 571.201 | 594.275 | 1.611 | 4.16 | 520.998 |
| 100 | 2000 | 0.76604 | 16.54 | 72.498 | 32.699 | 34.288 | 556.077 | 583.096 | 1.469 | 4.19 | 487.042 |
| 100 | 3000 | 0.77864 | 16.09 | 87.243 | 36.513 | 38.686 | 527.208 | 558.591 | 1.233 | 4.25 | 406.822 |
| 100 | 4000 | 0.79107 | 15.58 | 98.944 | 40.196 | 42.816 | 500.069 | 532.667 | 1.046 | 4.32 | 313.548 |
| 200 | 298.15 | 0.65634 | 8.84 | 13.521 | 19.845 | 19.949 | 998.100 | 1003.329 | 1.988 | 3.72 | 1050.163 |
| 200 | 500 | 0.65765 | 10.63 | 25.223 | 24.637 | 24.889 | 991.035 | 1001.142 | 1.918 | 3.73 | 1046.194 |
| 200 | 1000 | 0.66132 | 11.28 | 44.041 | 28.725 | 29.284 | 974.578 | 993.538 | 1.725 | 3.74 | 1028.463 |
| 200 | 2000 | 0.66873 | 10.93 | 66.039 | 33.566 | 34.593 | 943.786 | 972.691 | 1.401 | 3.77 | 972.465 |
| 200 | 3000 | 0.67589 | 10.36 | 80.963 | 37.928 | 39.286 | 914.987 | 947.743 | 1.152 | 3.80 | 898.619 |
| 200 | 4000 | 0.68272 | 9.74 | 92.879 | 42.165 | 43.734 | 887.965 | 921.010 | 0.955 | 3.83 | 811.519 |
| 200 | 5000 | 0.68918 | 9.09 | 103.100 | 46.325 | 48.001 | 862.619 | 893.828 | 0.796 | 3.85 | 713.419 |
| 328.9 | 298.15 | 0.59039 | 5.62 | 9.668 | 17.102 | 17.157 | 1460.662 | 1465.392 | 1.932 | 3.48 | 1595.617 |
| 328.9 | 500 | 0.59120 | 7.52 | 20.349 | 23.484 | 23.650 | 1453.220 | 1463.473 | 1.876 | 3.48 | 1592.551 |
| 328.9 | 1000 | 0.59359 | 8.31 | 38.750 | 28.759 | 29.160 | 1436.153 | 1456.210 | 1.680 | 3.49 | 1577.404 |
| 328.9 | 2000 | 0.59849 | 8.03 | 60.874 | 34.315 | 35.055 | 1404.771 | 1435.065 | 1.342 | 3.51 | 1526.660 |
| 328.9 | 3000 | 0.60317 | 7.51 | 76.059 | 39.179 | 40.135 | 1375.596 | 1409.158 | 1.084 | 3.52 | 1457.853 |
| 328.9 | 4000 | 0.60754 | 6.93 | 88.272 | 43.896 | 44.968 | 1348.294 | 1381.232 | 0.881 | 3.54 | 1375.511 |
| 328.9 | 5000 | 0.61158 | 6.33 | 98.811 | 48.541 | 49.646 | 1322.745 | 1352.852 | 0.719 | 3.55 | 1281.861 |
| 328.9 | 6000 | 0.61527 | 5.71 | 108.267 | 53.138 | 54.206 | 1298.874 | 1324.965 | 0.586 | 3.57 | 1178.247 |

Table S4. Thermodynamics properties of liquid Fe.

| *P*, GPa | *T*, K | *x=V/V*_0_ | αE–6,  K^-1^ | *S* | *C_V_* | *C_P_* | *K_T_* | *K_S_* | γ_th_ | *K*' | *G*,  kJ mol^-1^ |
| --- | --- | --- | --- | --- | --- | --- | --- | --- | --- | --- | --- |
|  |  |  |  | J mol^-1^K^-1^ | | | GPa | |  |  |  |
| 0.0001 | 1811 | 1.00000 | 87.88 | 99.664 | 33.844 | 43.159 | 83.701 | 106.737 | 1.729 | 5.97 | -100.203 |
| 0.0001 | 2000 | 1.01783 | 99.73 | 104.130 | 34.943 | 47.024 | 74.991 | 100.916 | 1.733 | 6.18 | -119.463 |
| 0.0001 | 2200 | 1.04000 | 116.69 | 108.848 | 36.167 | 52.335 | 65.217 | 94.371 | 1.741 | 6.48 | -140.760 |
| 10 | 2000 | 0.92217 | 56.23 | 98.473 | 34.089 | 40.181 | 131.285 | 154.746 | 1.589 | 5.23 | -42.695 |
| 10 | 2200 | 0.93302 | 60.91 | 102.414 | 35.124 | 42.592 | 123.226 | 149.426 | 1.587 | 5.32 | -62.786 |
| 50 | 2000 | 0.76459 | 22.79 | 89.327 | 32.643 | 34.640 | 316.053 | 335.385 | 1.342 | 4.25 | 221.366 |
| 50 | 2500 | 0.77377 | 25.02 | 97.372 | 34.729 | 37.619 | 299.860 | 324.814 | 1.330 | 4.30 | 174.644 |
| 50 | 3000 | 0.78400 | 27.58 | 104.514 | 36.854 | 40.882 | 282.835 | 313.743 | 1.321 | 4.35 | 124.143 |
| 100 | 2000 | 0.67684 | 13.69 | 84.338 | 31.811 | 32.856 | 517.595 | 534.597 | 1.200 | 3.86 | 506.323 |
| 100 | 3000 | 0.68687 | 15.76 | 98.527 | 35.519 | 37.505 | 487.999 | 515.294 | 1.183 | 3.90 | 414.568 |
| 100 | 4000 | 0.69859 | 18.17 | 110.006 | 39.301 | 42.647 | 455.754 | 494.553 | 1.171 | 3.95 | 310.150 |
| 200 | 2000 | 0.58520 | 7.86 | 79.220 | 30.914 | 31.423 | 884.008 | 898.559 | 1.047 | 3.51 | 1004.319 |
| 200 | 3000 | 0.59010 | 8.82 | 92.654 | 34.159 | 35.099 | 857.554 | 881.148 | 1.040 | 3.53 | 918.053 |
| 200 | 4000 | 0.59563 | 9.85 | 103.270 | 37.396 | 38.919 | 828.889 | 862.663 | 1.034 | 3.55 | 819.928 |
| 200 | 5000 | 0.60186 | 10.97 | 112.385 | 40.680 | 42.981 | 797.980 | 843.102 | 1.031 | 3.57 | 712.007 |
| 328.9 | 2000 | 0.51996 | 5.18 | 75.640 | 30.255 | 30.548 | 1321.218 | 1334.018 | 0.936 | 3.29 | 1568.188 |
| 328.9 | 3000 | 0.52281 | 5.76 | 88.629 | 33.194 | 33.730 | 1296.773 | 1317.733 | 0.936 | 3.30 | 1485.722 |
| 328.9 | 4000 | 0.52598 | 6.35 | 98.771 | 36.082 | 36.941 | 1270.307 | 1300.542 | 0.936 | 3.31 | 1391.857 |
| 328.9 | 5000 | 0.52950 | 6.98 | 107.368 | 38.984 | 40.260 | 1241.816 | 1282.460 | 0.937 | 3.32 | 1288.689 |
| 328.9 | 6000 | 0.53339 | 7.65 | 115.012 | 41.920 | 43.727 | 1211.281 | 1263.481 | 0.939 | 3.34 | 1177.436 |
